# Highly Efficient Degradation of Tetracycline Hydrochloride in Water by Oxygenation of Carboxymethyl Cellulose-Stabilized FeS Nanofluids

**DOI:** 10.3390/ijerph191811447

**Published:** 2022-09-11

**Authors:** Hong Xiao, Yingjun Wang, Hong Peng, Ying Zhu, Dexin Fang, Ganxue Wu, Li Li, Zhenxing Zeng

**Affiliations:** College of Environmental Sciences, Sichuan Agriculture University, Chengdu 611130, China

**Keywords:** tetracycline hydrochloride, CMC-FeS nanofluid, oxygenation, hydroxy radicals

## Abstract

The transformation of organic pollutants by stabilized nano-FeS in oxic conditions is far less understood than in anoxic states. Herein, carboxymethyl cellulose-stabilized FeS (CMC-FeS) nanofluids are prepared at a CMC-to-FeS mass ratio of 1/2 and their performance of tetracycline hydrochloride (TC) degradation under oxic conditions was investigated. Here, we showed that TC could be efficiently removed by oxygenation of CMC-FeS nanofluids at neutral initial pH. We found that CMC-FeS dosages as low as 15 mg/L can achieve the TC removal efficiency as high as 99.1% at an initial TC concentration of 50 mg/L. Oxidative degradation plays a predominated role in TC removal (accounting for 58.0%), adsorption has the second importance (accounting for 37.0%), and reduction has minor impact (accounting for 4.1%) toward TC removal. Electron spin resonance assays, fluorescent detection using coumarin as a probe, and radical scavenging experiments confirm that hydroxy radicals (•OH), both in free and surface-bound forms, contribute to oxidation of TC. Humic acids brought detrimental effects on TC removal and therefore should be biologically degraded in advance. This work offers a facile and cost-effective solution to decontaminate TC in natural and engineered water bodies.

## 1. Introduction

Tetracycline hydrochloride (TC) is one of the most commonly used antibiotics due to its inexpensive, convenient and excellent antibacterial properties [1]. In surface water or groundwater, TC concentrations are at levels of mg/L or even ng/L, whereas the TC concentration may be much higher in wastewaters. Up to 20 mg/L of TC has been detected in liquid manure [2]. In the effluent from a wastewater treatment plant serving about 90 bulk drug manufacturers, up to 31 mg/L of antibiotics has been reported [3]. As is known that TC is difficult to biodegrade owing to its biotic resistance and chemical stability, posing a huge challenge to natural environment and human health [4,5,6]. So far, the main methods to remove TC in water include advanced oxidation [7], biodegradation [8,9], membrane technology [10], and adsorption [11,12]. Unfortunately, low removal efficiency or high cost hindered their applications.

Recently, FeS has proved to be capable of effectively transforming chlorinated organic compounds (e.g., trichloroethane, trichloroethylene, and p-chloroaniline), nitroaromatic compounds, and polychlorinated biphenyls under anoxic conditions [13,14]. In addition, FeS has been reported to oxidatively degrade organic contaminants in the presence of oxygen. Cheng et al. [15] found that phenol could be efficiently oxidized in an oxic FeS suspension at circumneutral pH and hydroxyl radical (•OH) was the predominant reactive oxidant. Another recent study demonstrated that benzoic acid could be mainly degraded into 2,5-dihydroxybenzoic acid and the authors hypothesized that the strong oxidant must be a surface-localized oxidant with reactivity differing from those formed in free solution [16]. It is therefore concluded that oxygenation of FeS can trigger the oxidative degradation of organic contaminants. However, the reactive oxidants accounting for the oxidation has not been fully elucidated. •OH, singlet oxygen (^1^O_2_), superoxide radical (O_2_^•−^), sulfate radical (SO_4_^•−^), and Fe(IV) all fall into the category of reactive oxidants. As is known that Fe(II) can sequentially activate O_2_ to generate O_2_^•−^ and H_2_O_2_ conforming to the Haber–Weiss mechanism [17]. Hitherto, Wang et al. [18] proved that H_2_O_2_ could not directly oxidize TC and the impact of superoxide radical (O_2_^−^) on TC degradation could be ruled out. In addition, He et al. [16] presented evidence that singlet oxygen (^1^O_2_), sulfate radical (SO_4_^−^), and Fe(IV) could not be oxidants generated during FeS oxygenation under circumneutral conditions. Thereupon, the concern of oxidants is pointed to the types of hydroxyl radical, i.e., feely diffusing homogeneous or surface-bound •OH.

Compared to bulk particles or natural minerals, nanoscale FeS particles are expected to offer much greater sorption capacity and potentially greater reactivity due to their larger specific surface area [19]. Unfortunately, nanoparticles tend to agglomerate due to high surface energy and electrostatic attraction, which greatly reduced their advantages and therefore necessitated stabilization. Among varieties of stabilizers reported as yet, carboxymethyl cellulose (CMC) is one of the best candidates, which is not only cost-effective, but also “green” [20].

In this work, CMC-stabilized FeS (CMC-FeS) nanoparticles were prepared. CMC-FeS nanoparticles used here were in the form of suspensions, namely CMC-FeS nanofluids. To our best knowledge, degradation of TC by oxygenation of CMC-FeS nanofluids has never been reported. The main goals of this study are (1) to evaluate the performance of TC degradation by oxygenation of CMC-FeS nanofluids at low dosages and to constrain the reaction conditions under which significant TC oxidation occurs; (2) to ascertain the TC removal mechanism and the types of hydroxyl radical; and (3) to analyze the impact of organic impurities on TC degradation. To reach these goals, we (1) prepared CMC-FeS nanofluids and varied the mass ratio of CMC to FeS, dosage of CMC-FeS, initial pH as the main parameters of the reaction system; (2) conducted quenching experiments, electron spin resonance (ESR) assays, and fluorescent detection using coumarin as a probe to identify the predominant reactive oxidant; and (3) used humic acid (HA) as a representative of organic impurities to analyze its effect on TC degradation. This work provides a promising strategy to eliminate TC in both natural and engineered systems.

## 2. Materials and Methods

### 2.1. Chemicals

TC (98%) and CMC (MW = 90,000, in the sodium form, degree of substitute = 0.7) were purchased from Aladdin Chemistry Co., Ltd. (Shanghai, China). Ferrous sulfate heptahydrate (FeSO_4_·7H_2_O), sodium sulfide nonahydrate (Na_2_S·9H_2_O), sodium hydroxide (NaOH), hydrochloric acid (HCl), n-butanol, and potassium iodide (KI) were obtained from Chengdu Kelong chemical reagent company (Sichuan, China). 5,5-dimethyl-1-pyrroline-N-oxide (DMPO), 2-propanol, and coumarin were obtained from Sigma-Aldrich (St. Louis, MO, USA). All chemicals and reagents were analytically pure and used without further purification. Deionized water from a Heal Force NW ultrapure water system was used for all experiments.

### 2.2. Preparation of CMC-FeS Nanofluids

FeS particles were prepared by reacting Fe(NH_4_)_2_(SO_4_)_2_·6H_2_O (Mohr’s salt) with Na_2_S·9H_2_O at room temperature (25 °C). Mohr’s salt is the preferred reagent with ferrous iron since it is relatively resistant to oxidation [21]. The procedures to prepare CMC-FeS nanofluids were similar to those described by Liu et al. [22]. The CMC stock solution (1%, *w*/*w*) was prepared by dissolving CMC with deionized water and the solution was purged with purified N_2_ (>99%) for half an hour to clean up dissolved oxygen (DO). Stock solutions of 0.24 M Fe(NH_4_)_2_(SO_4_)_2_ and 0.24 M Na_2_S were also prepared with N_2_-purged deionized water. The Fe(NH_4_)_2_(SO_4_)_2_ solution was mixed with the CMC solution for half an hour to form Fe^2+^-CMC complexes in a 250 mL flask at room temperature (25 °C). Then, the Na_2_S solution was slowly introduced into the Fe^2+^-CMC solution for 10 min to yield the CMC-FeS nanoparticles. N_2_ was continuously supplied to purge out DO and provide mixing during the aforementioned reaction processes. CMC-FeS nanoparticles were used in the form of suspension, which were called CMC-FeS nanofluids. Subsequent filtering and drying processes were omitted, which minimized the reactivity loss of the nanoparticles due to possible contact with oxygen. CMC-FeS nanofluids with specific mass ratios of CMC to FeS (1:1, 1:2 and 1:5) were obtained by using pre-calculated volumes of stock solutions of CMC, Fe(NH_4_)_2_(SO_4_)_2_ and Na_2_S. Different dosages of CMC-FeS (calculated as mass concentrations of FeS) were achieved by adding specific volumes of CMC-FeS nanofluids. In fact, the preparation of non-stabilized FeS suspensions followed the same procedures except no CMC was used. The CMC-FeS nanoparticles were characterized or used within 1 h of preparation.

### 2.3. TC Removal in Oxic FeS Suspensions or CMC-FeS Nanofluids

TC removal experiments were conducted in conical flasks placed in a thermostatic oscillator (150 rpm, 25 ± 1 °C). The conical flasks were wrapped with aluminum foil to avoid any potential photochemical reactions and were exposed to air. TC concentration was set at 50 mg/L. pH was adjusted by 1 M NaOH and HCl. Predetermined dosages of CMC-FeS or FeS in the form of suspension were added and immediately reacted with TC. Then, 1.0 mL supernatant was collected at a certain time interval and filtered with a 25 nm membrane filter (Millipore Corp., Billerica, MA, USA) to determine the residual TC concentration.

To determine the optimal reaction conditions, batch experiments were conducted to explore the effects of initial pH (3.0, 7.0, 9.0), and CMC-FeS dosage (5, 10, 12.5, 15, 20 mg/L) on TC removal. The TC removal efficiency was calculated by Equation (1):

TC removal efficiency = (1 − *C_t_*/*C*_0_) × 100% (1)

where *C*_0_ (mg/L) and *C_t_* (mg/L) are the TC concentration at the initial time and at time *t*, respectively.

To confirm the production of •OH, ESR assays were carried out. To distinguish the free •OH and surface-bounded •OH, coumarin as a probe was used to confirm the formation of surface-bound •OH and quench experiments respectively using n-butanol and KI as scavengers were conducted. To clarify the contribution ratio of adsorption, oxidation and reduction in TC removal under the optimal conditions, the following experiments were designed: (1) total TC removal (*R*_total_): all the content after 60 min of reaction was filtered through a 25 nm membrane filter, and the filtrate was analyzed for residual TC. To prevent oxidation of TC, filtration was performed in an anaerobic glove box. (2) TC removal by adsorption (*R*_adsorption_): the filtration residue was further dissolved by 0.1 M HCl, thereafter repeatedly washed by 1 M NaOH, and the solute was analyzed for desorbed TC. (3) TC removal by oxidation (*R*_oxidation_): n-butanol was chosen as •OH scavenger, and differential value of TC removal with and without n-butanol represented the contribution of oxidation. (4) TC removal by reduction oxidation (*R*_reduction_) can be calculated as in Equation (2):

*R*_reduction_ = *R*_total_ − *R*_adsorption_ − *R*_oxidation_ (2)

To determine the mineralization rate of TC, Total organic carbon (TOC) removal was investigated. To eliminate the impact of adsorption on TOC degradation, thorough desorption of TC in the residue should be conducted as described above. All the experiments were repeated three times.

### 2.4. Analyses

TC was analyzed by a high-performance liquid chromatography (Agilent Technologies, 1260 Infinity) equipped with a C18 column (Agilent ZORBAX SB-C18, 5 μm, 4.6 mm × 250 mm). A total of 0.1 M oxalic acid, methanol (HPLC-grade) and acetonitrile (HPLC-grade) were used as mobile phase (V_oxalic acid_:V_methanol_:V_acetonitrile_ = 67:11:22) at the flow rate of 1.0 mL/min. The detection was performed with a UV detector at 360 nm wavelength with a detention time of 4.0 min. TOC was measured by a TOC analyzer (TOC-V CPH, Shimadzu, Tokyo, Japan). DO was detected by a portable dissolved oxygen meter (HQ30d, HACH, Loveland, CO, USA). pH was determined by a pH meter (INESA, PHSJ-5, Shanghai, China). The crystal structures of solids were observed by an X-ray powder diffractometer (XRD) (NOVA 2200E, Quantachrome, Boynton Beach, FL, USA). The valence state and composition of solids before and after the reaction were analyzed by an X-ray photoelectron spectroscopy (XPS) (Semerfly Technology Co., Ltd. Thermo Fisher Scientific, Waltham, MA, USA). The surface morphology of non-stabilized FeS particles and CMC-FeS nanoparticles after freeze-drying were characterized by scanning electron microscope (SEM) (JSM-7500F, JEOL, Tokyo, Japan). The morphology of non-stabilized FeS particles and CMC-FeS nanoparticles was observed by transmission electron microscopy (TEM) (F20, FEI, Hillsboro, OR, USA). The surface functional groups of CMC and the prepared particles were determined by Fourier transform infrared spectroscopy (FT-IR) (Nicolet IS5, Thermo Fisher Scientific, USA). The N_2_ adsorption–desorption isotherms were measured with an accelerated surface area and porosimetry system (Autosorb 1, Quatachrome) in order to determine the surface areas and pore volumes. Prior to the measurements, the samples were outgassed at 423 K under nitrogen flow for 4 h. The nitrogen adsorption–desorption data were recorded at liquid nitrogen temperature (77 K) and were measured over a relative pressure (*P*/*P*_0_) range from approximately 10^−6^ to 1. The BET surface area was calculated using the BET (Brunauer, Emmett and Teller) equation from the selected N_2_ adsorption data within the range of relative pressure, *P*/*P*_0_, from 0.1 to 0.3 [23]. The pH of the point of zero charge (pH_pzc_) of CMC-FeS nanoparticle was determined in accordance to the literature [24,25]. ESR assays were conducted according to the literature [15]. In order to capture as many •OH radicals as possible, 5.28 g/L CMC-FeS was oxygenated for 10 min first, to attain the stage of quick •OH production, and then, a 950 μL sample was withdrawn and immediately mixed with 100 μL of 1.47 M DMPO to form a DMPO-radical adduct. The mixture was shaken for 5 min and then measured on a JESFA200 spectrometer (JEOL, Japan) with a microwave bridge (receiver gain, 5020; modulation amplitude, 2 G; microwave power, 3 mW; modulation frequency, 100 kHz; center field: 326 mT). In the control experiments, the reaction conditions were the same as described above but under anoxic condition or with addition of 2 M 2-propanol. Fluorescent detection of surface-bound •OH using coumarin as a probe was fulfilled according to Leandri et al. [26]. The Fe^2+^ content was directly determined by the ferrozine (FZ) method [27]. To quantify the total aqueous Fe, hydroxylamine hydrochloride was used to reduce ferric ion (Fe^3+^) to Fe^2+^.

## 3. Results and Discussion

### 3.1. Characterizations

To characterize the physical stability of FeS nanoparticles under different CMC-to-FeS mass ratios, particle settling tests were conducted by monitoring the total Fe in the supernatants after the suspension let stand for 24 h. The results showed that 96.5% of particles settled without CMC and this percentage value became 32.5% at a CMC-to-FeS mass ratio of 1/5. Further, when the mass ratio was increased to 1/2 or higher, the particles were fully stabilized (i.e., 100% of particles were suspended). Thus, 1/2 was regarded as the minimum CMC-to-FeS mass ratio to fully stabilize the nanoparticles. Given that an appropriate amount of CMC can effectively prevent agglomeration of nanoparticles [28] whereas excessive CMC may hinder the contact between the active site on the surface of FeS particles and TC [29,30], the optimal CMC-to-FeS mass ratio was determined as 1/2.

**Figure 1:**
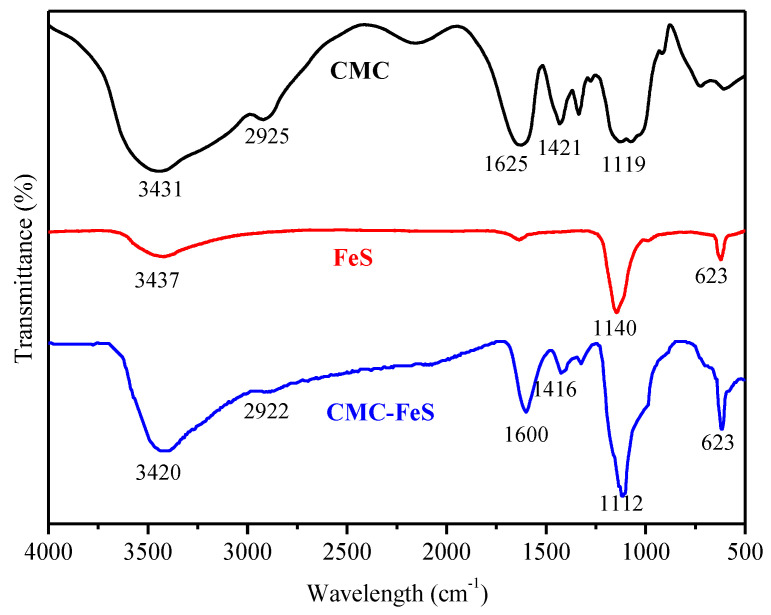
FT-IR spectra of pristine CMC (the black line), non-stabilized FeS (the red line), and CMC-FeS (the blue line). FT-IR spectra elucidate the chemical functional group of the characterized materials.

To convince the binding of CMC to FeS particles, the functional groups of pristine CMC, non-stabilized FeS and CMC-FeS were investigated by the FT-IR spectra (Figure 1). Bands at 3431, 2925, 1625, 1421, and 1119 cm^−1^ in the FTIR spectrum of CMC corresponded to the O−H stretch of carboxylic acid, asymmetric CH_2_ stretch, asymmetric stretching of COO^−^ group, symmetric stretching of COO^−^ group, and O−H deformation, respectively. The FTIR spectra of naked FeS show bands at 3437 cm^−1^, which may be attributed to absorption by residual H_2_O. Absorption bands at 1140 and 623 cm^−1^ indicate that Na_2_SO_4_ remained in the sample. In the FTIR spectra of CMC-FeS, bands corresponding to the O−H stretch of carboxylic acid, asymmetric CH_2_ stretch, asymmetric and symmetric stretching of COO^−^ groups shifted to 3420, 2922, 1600, and 1416 cm^−1^, respectively. Based on previous researches [31,32], the interaction between the carboxylate head and the metal atom could be categorized into four types, i.e., monodentate, bridging bidentate, chelating bidentate, and ionic interaction, which can be diagnosed by the wavenumber separation (Δ) between the ν_as_(COO^−^) and ν_s_(COO^−^). The Δ of 200–300 cm^−1^ indicates monodentate chelation, Δ < 100 cm^−1^ suggests bidentate chelation, and Δ of 140–190 cm^−1^ hints bidentate bridging [33]. In present study, the Δ between ν_as_(COO^−^) (1600 cm^−1^) and ν_s_(COO^−^) (1416 cm^−1^) is 184 cm^−1^ in the FTIR spectrum of CMC-FeS, which suggests that the primary mechanism for binding CMC to FeS is bidentate bridging [34]. Additionally, the O−H stretch band shifted from 3431 cm^−1^ for pure CMC to 3420 cm^−1^ for CMC-FeS, which was ascribed to enhanced intermolecular hydrogen bonding between CMC and FeS [35]. The FTIR results suggest that the stabilization of FeS particles is facilitated through adsorption of CMC molecules onto the surface of the nanoparticles via carboxylate and hydroxyl groups. The partial encapsulation of the nanoparticles with negatively charged CMC induces a strong negative potential that prevents aggregation of the like particles. The CMC-induced negative potential was revealed by the highly negative ζ potential (−43 to −68 mV) over the pH range of 5−11 [22]. Therefore, CMC stabilizes the nanoparticles through concurrent electrostatic repulsion and steric hindrance.

**Figure 2:**
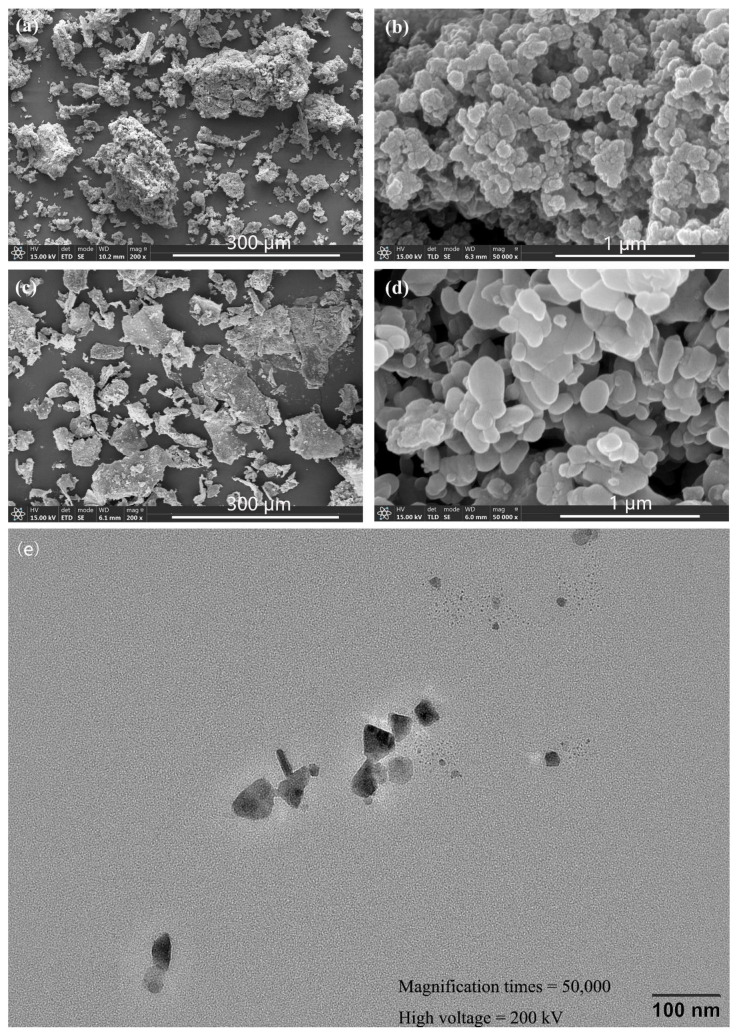
SEM images of FeS under 200× (**a**), FeS under 50,000× (**b**), CMC-FeS under 200× (**c**), CMC-FeS under 50,000× (**d**), and TEM images of CMC-FeS nanoparticles (**e**). SEM images elucidate microscopic morphology and size of the characterized particles. TEM images can be analyzed by a specialty image processing software named ImageJ to give the particle size distribution.

Figure 2 shows the SEM images of the freeze-dried FeS and CMC-FeS, and the TEM image of CMC-FeS nanoparticles.

Obviously, the non-stabilized FeS particles stacked together and appeared as micrometer-sized aggregates, whereas the CMC-FeS particles were evenly dispersed and maintained nano-level size. TEM analysis revealed that CMC-FeS particles were with a size spanning from 24.95 to 62.13 nm and a mean size of 46.31 ± 10.46 nm. In addition, the textural properties (Table S1) characterized by N_2_ adsorption–desorption isotherms showed that the BET surface area, pore size, and pore volume of CMC-FeS nanoparticles and FeS particles were 244.690 and 19.103 m^2^/g, 2.192 and 2.198 nm, and 0.049 and 0.070 mL/g, respectively. Noteworthily, the BET surface area of CMC-FeS nanoparticles was 12.8 times that of FeS particles, which is beneficial to the adsorption of TC.

**Figure 3:**
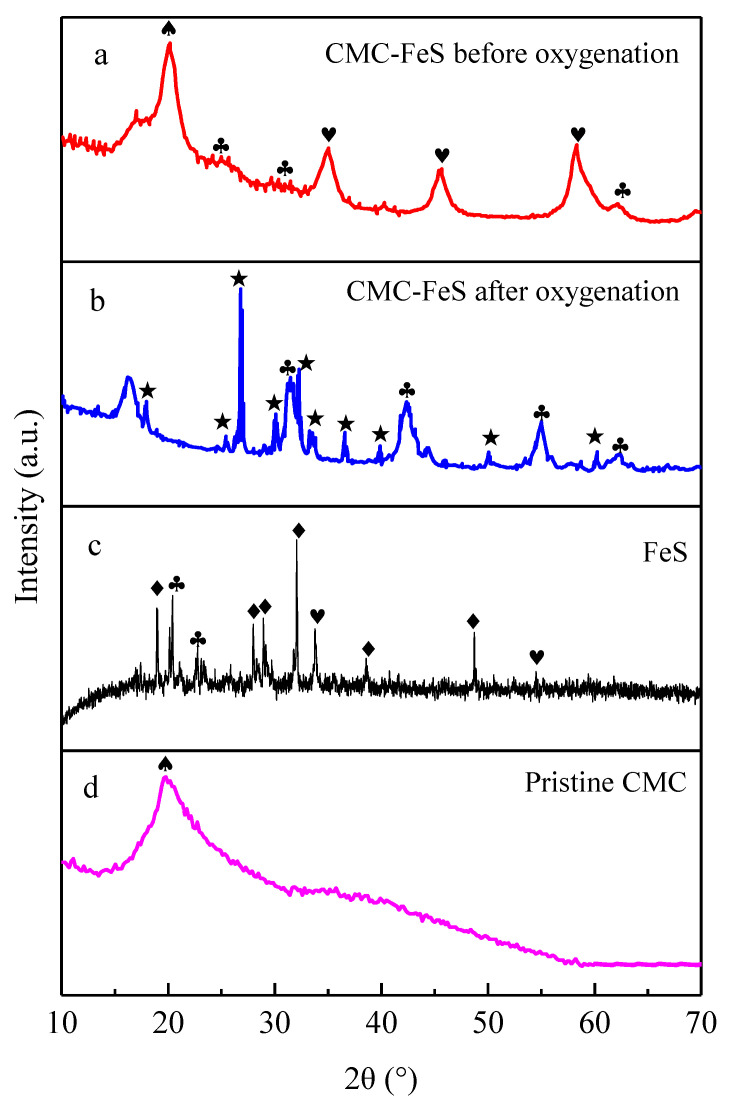
XRD patterns of pristine CMC (**a**), FeS (**b**), CMC-FeS before oxygenation (**c**), and CMC-FeS after oxygenation (**d**). ♣: FeOOH; ♦: Na_2_SO_4_; ♥: FeS; ♠: CMC; ★: Sulfur.

During the course of CMC-FeS oxygenation, the black suspension gradually transformed into a yellow one. The XRD results suggested that FeS was oxidized into elemental sulfur and FeOOH (Figure 3a or Figure 3b), in agreement with the findings of other researchers [16,17]. FeS was easily oxidized during its preparation and characterization (Figure 3c), whereas the involvement of CMC alleviated the degree of such oxidation (Figure 3a or Figure 3d).

**Figure 4:**
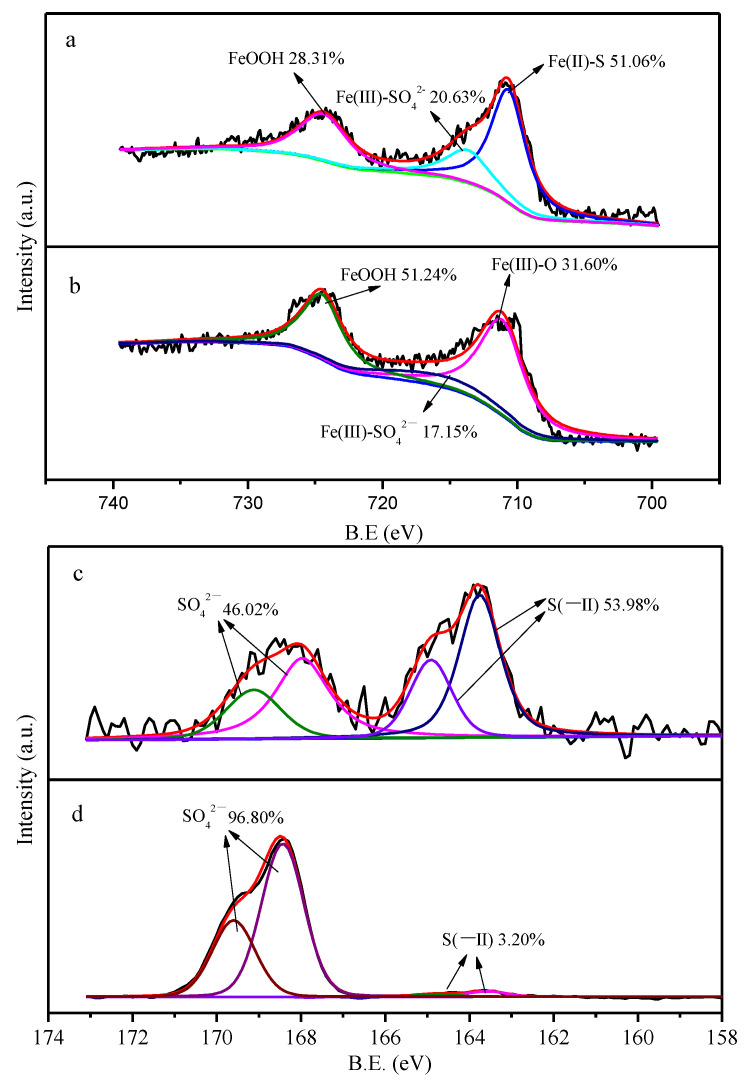
XPS spectra before and after reaction for TC Removal by CMC-FeS. Fe 2p before (**a**) and after (**b**); S 2p before (**c**,**d**).

In order to better understand the modifications on the surface of particles, an XPS analysis was implemented. The XPS spectra (Figure 4a) show that three peaks appeared in the spectra of Fe 2p before CMC-FeS oxygenation, which were FeOOH (28.31%), Fe(III)-SO_4_^2−^ (20.63%), and Fe(II)-S (51.06%) with the corresponding binding energy of 724.5, 713.0, and 710.6 eV, respectively [36,37]. After the reaction, the Fe 2p spectra (Figure 4b) changed and the peaks corresponded to FeOOH (51.25%), Fe(III)-SO_4_^2−^ (17.15%), and Fe(III)-O (31.60%), with the binding energy of 710.5, 713.2, and 723.8 eV, respectively. During the reaction, the proportion of Fe(II)-S decreased from 51.06% to 0%, while the FeOOH and Fe(III) increased. XPS results verified the oxidation of FeS to FeOOH, as demonstrated by XRD. As regards S species, XPS results showed that SO_4_^2−^ and S(−II) occupied 46.02% and 53.98%, respectively, before reaction (Figure 4c). The peaks of SO_4_^2−^ centered at 168.0 and 169.1 eV, and the peaks of S(−II) centered at 163.8 eV and 164.9 eV [38]. After reaction, the remained S(−II) whose peak centered at 163.6 eV and 164.8 eV only accounted for 3.20%, which implied a drastic oxidation of S(−II) (Figure 4d). Noteworthily, no elemental sulfur with peak centering at 164.0 eV was detected although elemental sulfur should have been produced as the main oxidation product of S(−II) [38]. The absence of elemental sulfur can be ascribed to its tiny content in the sample and unavoidable sublimation under vacuum conditions during XPS analysis [39].

### 3.2. TC Removal at Different CMC-FeS Dosages

**Figure 5:**
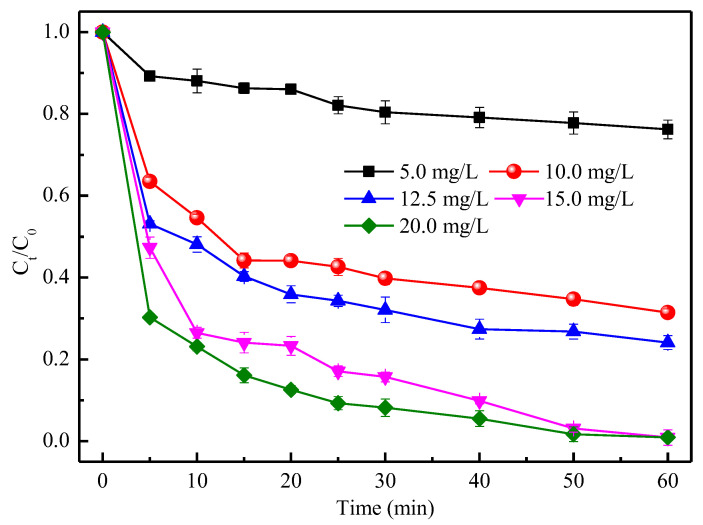
Effect of CMC-FeS dosage on TC removal. *C*_0_ (mg/L) and *C_t_* (mg/L) are the TC concentrations at the initial time and at time *t*, respectively. Conditions: *C*_0_ = 50 mg/L, CMC-to-FeS mass ratio = 1:2, Initial pH = 7.0; thermostatic oscillator (150 rpm, 25 ± 1 °C).

To ascertain the constraining conditions for TC removal, the effect of CMC-FeS dosage on TC removal under oxic condition was first investigated (Figure 5).

After 60 min of reaction, the TC removal efficiency was increased from 23.8% to 99.1% when the CMC-FeS dosage was initially increased from 5.0 to 15.0 mg/L. This is because an increase in CMC-FeS dosage will increase the number of active sites whether for oxidation or for adsorption. However, when further increasing CMC-FeS dosage from 15.0 to 20.0 mg/L, no further increase in the TC removal efficiency took place. This result indicated that a mass transfer limit existed between TC and CMC-FeS [15]. Another explanation is that low FeS contents produced less •OH but higher utilization efficiency, while high FeS contents produced more •OH but lower utilization efficiency [15]. In addition, if focusing on the TC removal profile during the initial phase of the reaction (especially, the initial 10 min), it was obvious that TC removal always became faster along with the increase of CMC-FeS dosage from 5 to 20 mg/L. This trend disagreed with the observation in a similar study conducted by Cheng et al. [15], who found that the initial phenol degradation rate increased with the increase in FeS dosage from 200 to 1000 mg/L and then decreased with further increase in FeS dosage to 5 g/L. Cheng et al. [15] asserted that DO might have become the limiting reaction partner during an initial phase of reaction and at higher FeS dosages. In the present study, the CMC-FeS dosage was set at lower levels (5–20 mg/L), DO replenished by oscillation (150 rpm) is sufficient for its reaction with FeS-bound Fe(II) and, therefore, no DO limit occurred. It should be noted that although CMC-FeS dosage (5–20 mg/L) in the present experiment was far less than the FeS dosage (200–1000 mg/L) used by Cheng et al. [15], an excellent removal performance was achieved. Herein, the distinguished reactivity of CMC-FeS nanofluids may have played a vital role. In sum, a CMC-FeS dosage of 15 mg/L can be reckoned as the optimal dosage in this study.

### 3.3. TC Removal at Different Initial pH Values

**Figure 6:**
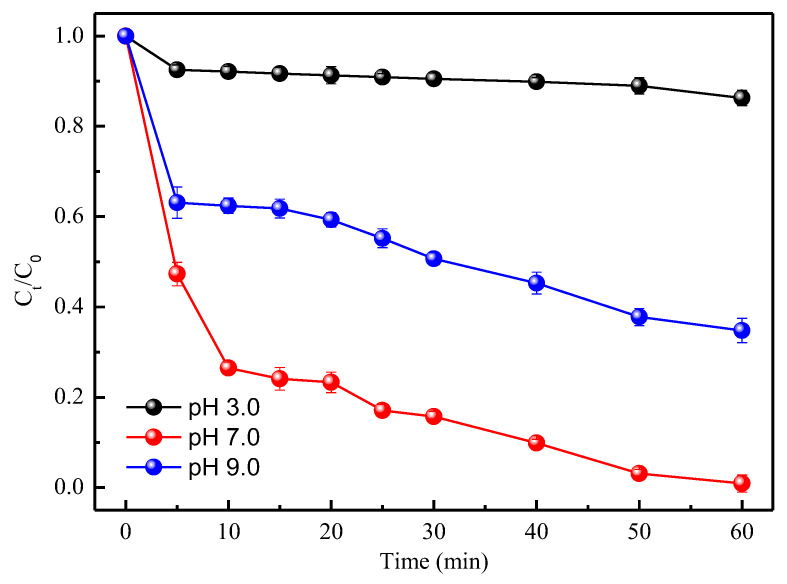
Effect of initial pH value on TC removal. *C*_0_ (mg/L) and *C_t_* (mg/L) are the TC concentrations at the initial time and at time *t*. Conditions: *C*_0_ = 50 mg/L; CMC-FeS dosage = 15 mg/L; CMC-to-FeS mass ratio = 1:2; thermostatic oscillator (150 rpm, 25 ± 1 °C).

Figure 6 depicts effects of the initial pH on TC removal by CMC-FeS. The removal efficiency received its minimum value (13.7%) at the initial pH 3.0 whereas achieved its maximum value (99.1%) at the initial pH 7.0. Further, the removal efficiency fell to 65.2% with pH increased to 9.0.

The TC removal pathways may include adsorption, oxidation, and reduction, which are all pH-dependent. The initial pH can significantly affect the surface properties of CMC-FeS nanoparticles, the chemical form of aqueous TC, and the generation of reactive oxygen species (ROS). For the initial pH 3.0, the corresponding lowest TC’s removal efficiency (13.7%) may be ascribed to two reasons: first, pH 3.0 is the lowest pH level in the present study. The more acidic is the aqueous solution, the more CMC-FeS nanoparticles are dissolved and, thus, the more reactive sites are lost [39,40]. As shown in Figure S1, CMC-FeS nanoparticles were almost completely dissolved at pH 3.0, while 31.5% of Fe was dissolved at pH 6.0 and the particle dissolution was negligible at pH ≥ 7.0. Tetracycline molecules can be predominantly expressed as positive ion state (H_3_L^+^) for pH < 3.3. H_3_L^+^ under such an acidic conditions is stable [41], and it is not easily complexed with metal ions [42]. When the initial pH was 7.0, the highest removal efficiency (99.1%) of TC could be explained as follows: first, the electrostatic repulsion between tetracycline molecules and CMC-FeS nanoparticles disappeared due to the neutral state (H_2_L) of tetracycline molecules at 3.3 < pH < 7.6 [43]. Second, FeS can produce •OH by oxygenation under circumneutral conditions [15,17]. Third, appropriate iron corrosion products are formed on the surface of FeS after reaction. Iron corrosion products show both positive and negative effects on the removal of TC [44]. On the one hand, ferric hydroxides, as the main iron corrosion products, possess a strong complexing effect with TC [45]. On the other hand, iron corrosion products may block the reaction sites on FeS nanoparticles. Fe(II) species on the surface has been proved to be crucial active sites of adsorption and reduction [46]. When the initial pH rises from 7.0 to 9.0, excessive iron corrosion products cause a severe blockage of the reaction sites on FeS nanoparticles. Even worse, the main form of the TC molecule at pH 9.0 turns into the negative ion state [43]. The complexation between negative HL^−^ and ferric hydroxide was restrained, which is incurred by electrostatic repulsion at pH 9.0. The surface of ferric hydroxide at pH 9.0 is inferred to be negatively charged based on that the pH_zpc_ of the ferric hydroxide mineral is 7.9 [47]. Likewise, the pH_pzc_ of CMC-FeS was determined as ~6.5, and therefore, the electrostatic repulsion also existed between HL^−^ and CMC-FeS at pH 9.0. More importantly, the reduced TC removal is related to the weak or even negligible oxidation functioning under the alkaline condition. Chen et al. [48] found that no 2,4-dichlorophenoxyacetic acid was degraded at the high pH value (pH = 8.0) after 240 min of reaction time by 0.5 g/L of FeS and 10 mM of H_2_O_2_.

### 3.4. Mechanisms of TC Removal

The TC removal pathways may include adsorption, oxidation, and reduction. In the present study, the three pathways respectively contributed 58.0% (oxidation), 37.0% (adsorption), and 4.1% (reduction) to the TC removal at the initial pH 7.0. Evidently, oxidative degradation played a predominant role, which was also supported by the relatively high TOC removal efficiency (53.6%) in the present study. In addition, for TC degradation, Cao et al. [49] reported a TOC removal efficiency of 15.6% by peroxymonosulfate activated with zero-valent iron (Fe^0^), Zhang et al. [50] reported a TOC removal efficiency of 48.7% in the natural bornite/persulfate system, and Cao et al. [46] achieved a TOC removal efficiency of 53.4% in the Fe^0^/air process.

To substantiate the role of •OH, electron spin resonance (ESR) assays, fluorescent detection using coumarin as a probe, and quenching experiments were conducted.

**Figure 7:**
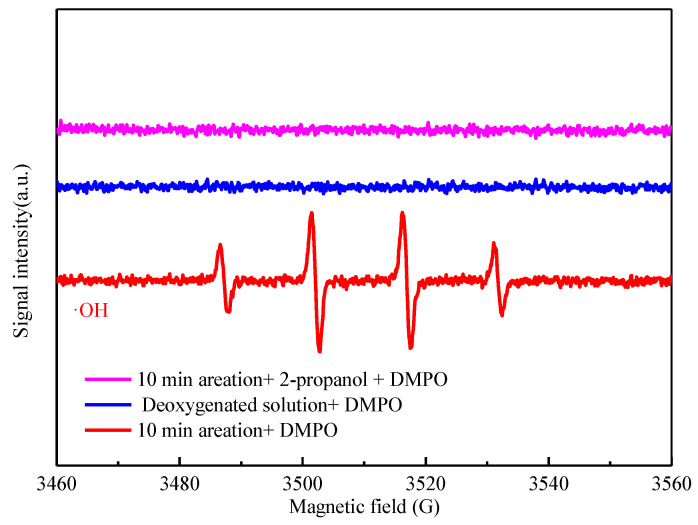
ESR spectra for •OH produced in different systems.

**Figure 8:**
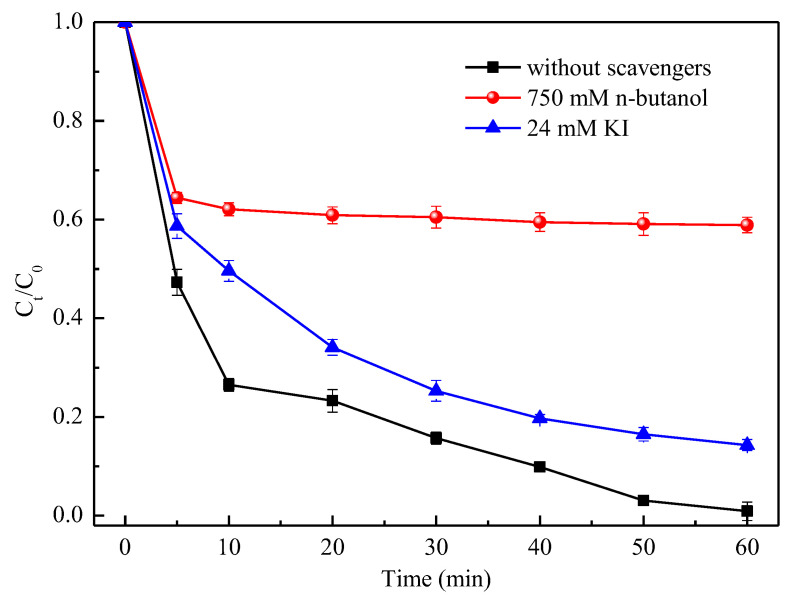
Effect of radical scavengers on TC removal. *C*_0_ (mg/L) and *C_t_* (mg/L) are the TC concentrations at the initial time and at time *t*, respectively. Conditions: *C*_0_ = 50 mg/L; CMC-FeS dosage = 15 mg/L; CMC-to-FeS mass ratio = 1:2; initial pH = 7.0; thermostatic oscillator (150 rpm, 25 ± 1 °C).

In the case of 60 mM CMC-FeS stock suspension at oxic condition, the ESR spectrum (Figure 7) shows the characteristic 1:2:2:1 peaks for •OH−DMPO adduct. In comparison, the signals for •OH−DMPO were not detected under anoxic condition or with addition of 2 M 2-propanol as an •OH scavenger (*k*_2-propanol,•OH_ = 1.9 × 10^9^ M^−1^·s^−1^) [51]. To further differentiate free •OH and surface-bound •OH, n-butanol and KI were used in the quenching experiments. Excess n-butanol can scavenge all the hydroxyl radicals produced (both surface-bound •OH and free •OH), whereas excess KI prefers to react with surface-bound •OH [48,52]. The difference in removal efficiencies linked with excess n-butanol and excess KI is attributed to free •OH. The results (Figure 8) clearly show that TC removal was greatly inhibited (TC removal efficiency decreased from 99.1% to 41.1% at 60 min) in the presence of 750 mM n-butanol, suggesting that TC was oxidized by •OH both on the surface of CMC-FeS nanoparticles and in the bulk liquid. With addition of excess KI (24 mM), the TC removal efficiency decreased from 99.1% to 85.7% at 60 min. The results of quenching experiments implied that 44.6% removal of TC might be attributed to the free •OH, meanwhile, 13.4% removal of TC was owed to the surface-bound •OH. 

To confirm the formation of surface-bound •OH more apparently, coumarin as a probe was used due to the unique fluorescent product 7-hydroxycoumarin formed in the reaction between surface-bound •OH and coumarin [53]. Fluorescent images (Figure S2) clearly demonstrated the presence of the surface-bound •OH. 

Our results proved that •OH can be produced from CMC-FeS oxidation under oxic conditions, agreeing with findings of previous research documenting that the interaction between FeS and O_2_ could generate •OH under dark and neutral conditions [17]. Referring to previous research [15,17,44,54,55], we summarized the mechanisms •OH production as follows: the interaction of O_2_ with structural ≡Fe^II^ on CMC-FeS surface forms a surface complex, and then, the complexed O_2_ is reduced to H_2_O_2(ad)_ via a two-electron transfer process. A portion of freshly generated H_2_O_2(ad)_ is then decomposed by FeS to produce surface-bound •OH (OH_(ad)_), and the other portion of H_2_O_2(ad)_ desorbs into the solution to be H_2_O_2(aq)_. Fe^2+^_(aq)_ leached from CMC-FeS particles reacts with the H_2_O_2(aq)_ to generate free •OH (OH_(aq)_) through the Fenton mechanism. It is noteworthy that both structural ≡Fe^X^ and Fe^X+^_(aq)_ can complex with TC. Herein, Fe(X) was used to denote either ≡Fe^X^ or Fe^X+^_(aq)_. During the reaction, a proportion of Fe(II) would be regenerated from the reduction of Fe(III) by FeS and S(−II), which was beneficial to •OH production [44]. As a whole, the mechanism of TC degradation upon oxygenation of CMC-FeS was proposed to consist of reactions shown in Equations (3)–(9).

Fe(II) + TC → Fe(II)-TC (3)

Fe(II)-TC + O_2_ → Fe(III)-TC + O_2_ (4)

Fe(II)-TC + O_2_^•−^ + H^+^ → Fe(III)-TC + H_2_O_2_ (5)

S(−II) + Fe(III) → Fe(II) + S(0) (6)

Fe(II)-TC + H_2_O_2_ → Fe(III)-TC + •OH + OH^−^ (7)

TC + •OH → degradation products (8)

Fe(II) + •OH → Fe(III) + OH^−^ (9)

After 60 min of reaction, pristine FeS was completely consumed and lepidocrocite (γ-FeO(OH)) was detected to be the only iron corrosion product, as demonstrated by He et al. [16]. Lepidocrocite, as a kind of ferric hydroxides, still has a strong complexing effect with TC [45], which is mainly responsible for TC removal by adsorption (37.0%). As regards TC removal by reduction, it can be largely ascribed to a reducing agent (*e_aq_*^−^). *e_aq_*^−^ is highly reactive towards molecules containing a carbonyl group adjacent to a double bond [56]. As the site of electron addition, the tricarbonyl group of TC reacts with *e_aq_*^−^ and generates semi-reduced TC, which is a strong reducing species capable of transferring electron to other byproducts. The minor portion of removal (4.1%) by reduction may resort to suppression incurred by oxidation and adsorption, which occupied a large number of carbonyl groups [46].

### 3.5. TC Removal in the Presence of Co-Existing Organic Impurities

**Figure 9:**
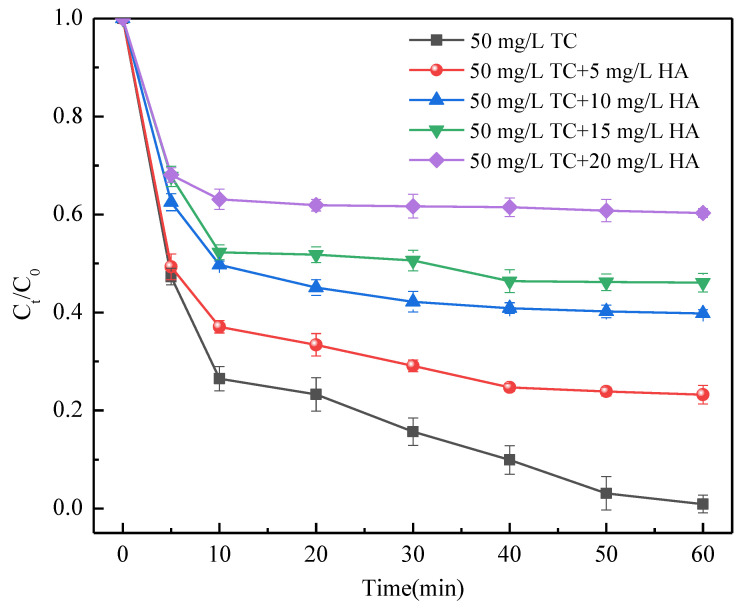
TC removal in the presence of TC and HA. *C*_0_ (mg/L) and *C_t_* (mg/L) are the TC concentrations at the initial time and at time *t*, respectively. Conditions: *C*_0_ = 50 mg/L; [HA] = 5, 10, 15, 20 mg/L; CMC-FeS dosage = 15 mg/L; CMC-to-FeS mass ratio = 1:2; initial pH = 7.0; thermostatic oscillator (150 rpm, 25 ± 1 °C).

In contaminated water, there are likely other organic matters besides TC. These organic matters may negatively affect the removal of TC. Humic acid (HA) is selected as representative because it is an ubiquitously dissolved organic matter in polluted water and has a high density of carboxylate functional groups that may complex with Fe(II) [57]. To assess the TC removal efficiency in the co-existence of TC and HA, different concentrations of HA were added into solution containing 50 mg/L of TC. Results (Figure 9) revealed that the TC removal efficiency decreased significantly with the increasing HA concentration. Compared with TC, organic matter such as HA was more susceptible to biodegradation. Therefore, it is recommended to use the biological treatment to remove the organic pollutants such as HA in advance as much as possible, alleviating their adverse effect on TC removal.

### 3.6. Environmental Implications

Although FeS has been extensively reported for reducing contaminants under anoxic conditions, its oxidizing effect under oxic conditions has been greatly underestimated and mostly limited to the natural environments. In this study, •OH was confirmed to be produced upon oxygenation of CMC-FeS, in both free and surface-bound forms. CMC was used to prevent agglomeration of nano-FeS particles and proved to be efficient at a low CMC-to-FeS mass ratio. Although CMC-FeS is nonreusable (Figure S3), excellent removal efficiency of TC and fairly good removal efficiency of TOC were achieved at a quite low dosage level of CMC-FeS, demonstrating its cost-effectiveness. The dosages of FeS in the oxygenation system reported elsewhere, whether 1 g/L [15] or 0.9 g/L [16], are too high to be cost-effective from the view of engineering applications. CMC-FeS nanofluids can be prepared easily on site and used conveniently just like adding traditional flocculants. The advantages mentioned above endow CMC-FeS nanofluids a brilliant prospect in engineering applications to treat industrial wastewater, domestic sewage, livestock and poultry breeding, and aquaculture wastewater. It is worth mentioning that other organic pollutants may compete with target biorecalcitrant pollutants such as TC for •OH, and therefore, biological pretreatment is strongly suggested. Besides organic matter, there are various inorganic ions (such as chloride, carbonate/bicarbonate, nitrate, and sulfate) in real wastewater. The effect of inorganic ions on TC removal is another issue deserving further research. Especially, while encountering combined pollution of heavy metal and biorecalcitrant organic matter, it is recommended to design a two-stage processing system operated under a sequence batch mode, which constitutes of one anoxic stage (dominated by reduction and adsorption) and another oxic stage (dominated by oxidation). In this regard, comprehensive investigations are needed in the future.

## 4. Conclusions

Highly efficient degradation of TC in water by oxygenation of CMC-FeS nanofluids was achieved in this study. The required dosage of CMC-FeS can be very low, which guarantees its cost-effectiveness. The initial pH has significant impact on TC removal and neutral pH is preferred. The TC removal pathways include adsorption, oxidation and reduction, and oxidation plays a pivotal role attributed to the production of free •OH and surface-bounded •OH. Co-existing organic impurities will impose a negative effect on TC removal and therefore necessitate biological pretreatment. This work proposes a facile strategy to remove TC under both natural and engineered scenarios.

## Supplementary Materials

The following supporting information can be downloaded at: https://www.mdpi.com/1660-4601/19/18/11447/s1, Figure S1: Dissolution of CMC-FeS measured as soluble Fe as a function of the initial pH. Experimental conditions: FeS dosage = 15 mg/L, temperature = 25 ± 1 °C. *M_dissolved_* is the mass of Fe in the aqueous phase, and *M*_0_ is the total mass of Fe. Figure S2: Fluorescent images of CMC-FeS nanofluids using coumarin as a probe. Blue fluorescence characterizes unique fluorescent product 7-hydroxycoumarin formed in the reaction between surface-bound •OH and coumarin. Conditions: [Coumarin] = 100 μM; excitation wavelength = 332 nm; magnification times = 1000; [CMC-FeS] = 5.28 g/L; CMC-to-FeS mass ratio = 1:2; initial pH = 7.0; CMC-FeS nanofluids subjected to air purging for 5 min before detection. Figure S3: Reusability of CMC-FeS nanofluids for TC removal. Table S1: Typical textural properties of FeS and CMC-FeS.
