# Supplementary material for: Highly Efficient Degradation of Tetracycline Hydrochloride in Water by Oxygenation of Carboxymethyl Cellulose-Stabilized FeS Nanofluids
Source: Int J Environ Res Public Health. 2022 Sep 11;19(18):11447. doi: 10.3390/ijerph191811447 (PMC9565224; doi:10.3390/ijerph191811447)
Supplement: Supplementary file 1 [file ijerph-19-11447-s001.zip › ijerph-1880953-supplementary.pdf]

Table S1. Typical textural properties of FeS and CMC-FeS.

	FeS	CMC-FeS
BET surface area (m^2/g)	19.103	244.690
External surface area (m^2/g)	19.103	52.924
Micropore surface area (m^2/g)	0	191.716
Total pore volume (mL/g)	0.070	0.049
Micropore volume (mL/g)	0	0.019
Mesopore volume (mL/g)	0.070	0.030
Average pore diameter (nm)	2.198	2.192

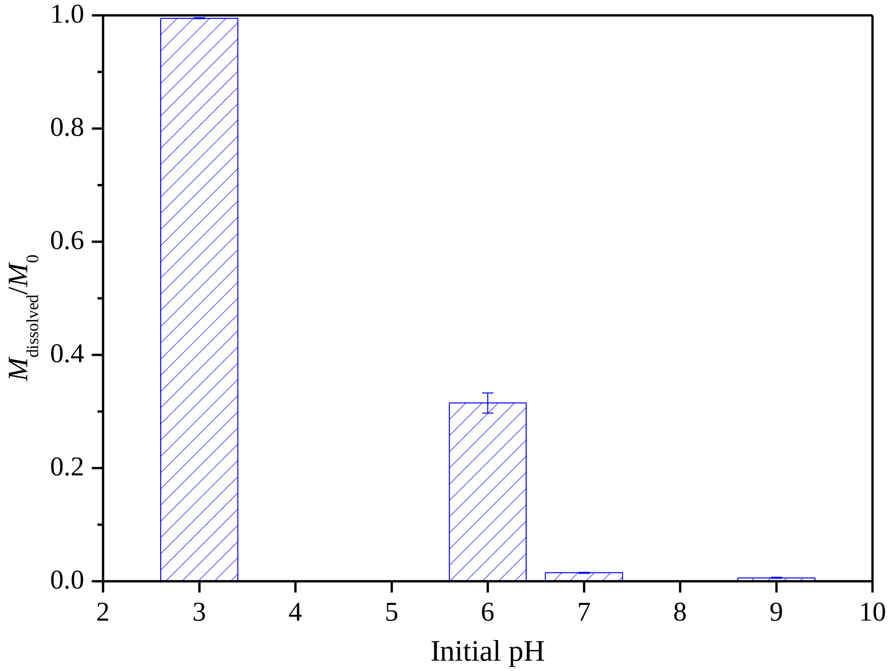

Figure S1. Dissolution of CMC-FeS measured as soluble Fe as a function of the initial pH. Experimental conditions: FeS dosage = 15 mg/L, temperature = 25 ± 1 °C. $M_{dissolved}$ is the mass of Fe in the aqueous phase, and M_0 is the total mass of Fe.

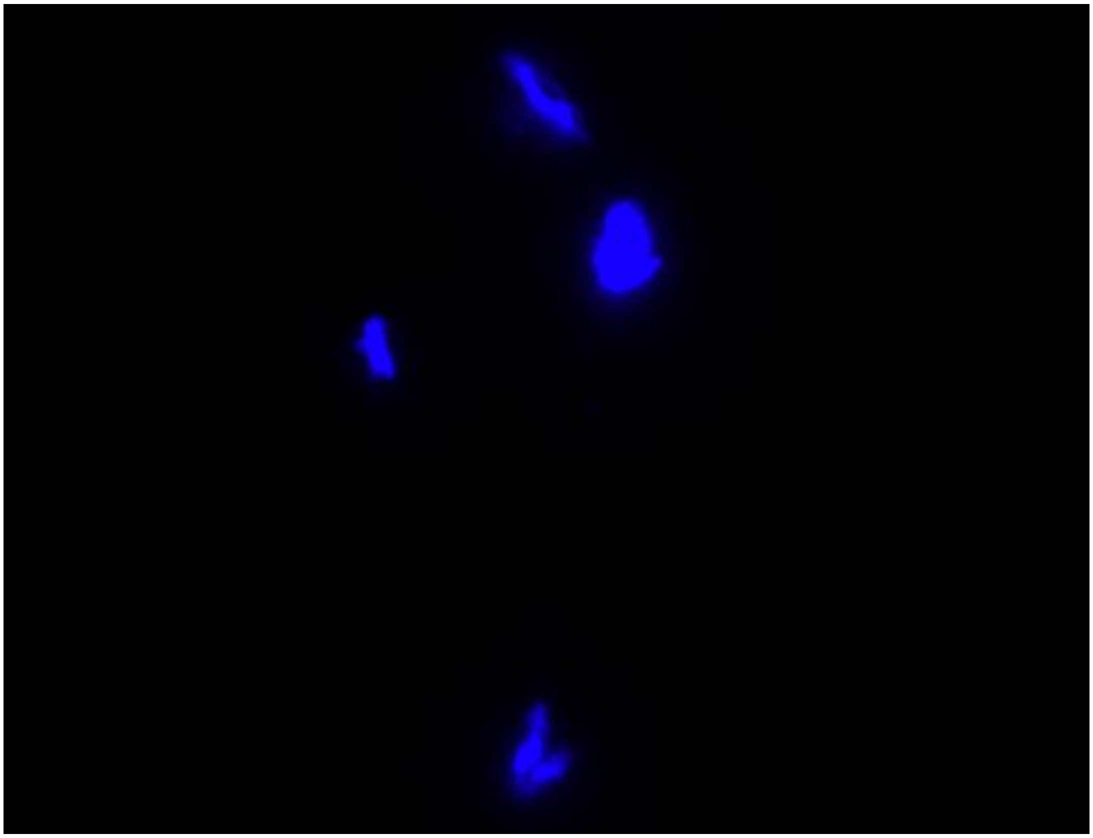

Figure S2. Fluorescent images of CMC-FeS nanofluids using coumarin as a probe. Blue fluorescence characterizes unique fluorescent product 7-hydroxycoumarin formed in the reaction between surface-bound •OH and coumarin. Conditions: [Coumarin] = 100 μM; Excitation wavelength = 332 nm; Magnification times = 1000; [CMC-FeS] = 5.28 g/L; CMC-to-FeS mass ratio = 1:2; Initial pH = 7.0; CMC-FeS nanofluids subjected to air purging for 5 min before detection.

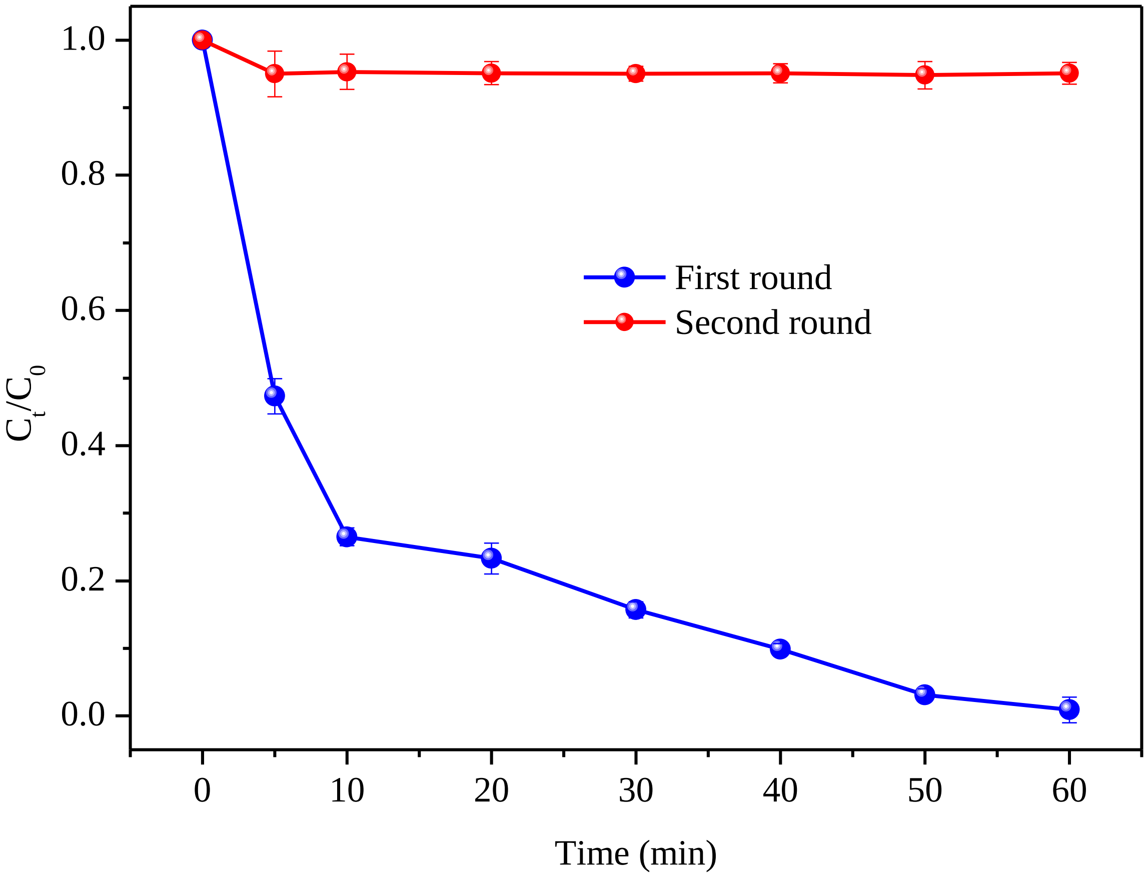

Figure S3. Reusability of CMC-FeS nanofluids for TC removal.
